# Supplementary material for: Beyond Platinum, ICIs in Metastatic Cervical Cancer: A Systematic Review
Source: Cancers (Basel). 2022 Dec 1;14(23):5955. doi: 10.3390/cancers14235955 (PMC9737392; doi:10.3390/cancers14235955)
Supplement: Supplementary file 1 [file cancers-14-05955-s001.zip › cancers-1956367-supplementary.pdf]

“Beyond platinum: ICIs in metastatic cervical cancer. A systematic review.”

SUPPLEMENTARY MATERIAL

Table S1. PICOS structure for study selection.

**P**atients	Adult women with cervical cancer in the recurrent/metastatic setting
**I**ntervention	Immune checkpoint inhibitors (ICIs)
**C**omparison	Not ICIs
**O**utcome(s)	Response rates (ORR, DCR), survival outcomes (PFS, OS), adverse events (number and grade)
**S**tudy Design	Prospective clinical trials

	Confounding bias	Selection bias	Classification intervention bias	Deviation from intended intervention	Missing data bias	Measure outcome bias	Selected outcome bias	Overall bias
Frenel et al (NCT02054806)[24]	+	+	+	+	+	?	+	+
Youn et al (NCT03444376)[25]	+	+	?	+	+	?	+	?
Chung et al (NCT02628067)[26]	+	+	+	+	+	+	+	+
Colombo et al (NCT03635567)[27]	+	+	+	+	+	+	+	+
Santin et al (NCT02257528)[28]	+	+	+	?	?	+	+	?
Naumann et al (NCT02488759)[29]	+	+	+	?	?	+	+	?
Oaknin et al (NCT02488759)[30]	+	+	+	?	?	+	+	?
Tamura et al (JapicCTI163212)[31]	+	+	+	+	+	?	+	+
O'Malley et al (NCT03104699)[32]	+	+	+	+	+	?	+	+
O'Malley et al (NCT03495882)[33]	+	+	+	+	+	?	+	+
Tewari et al (NCT03257267)[34]	+	+	+	+	+	+	+	+
Rischin et al (NCT02760498)[35]	+	+	+	+	+	?	+	+
Lan et al (NCT03816553)[36]	+	+	+	+	+	?	+	+
Zhang et al[37]	+	+	+	+	+	+	+	+
Zheng et al[38]	+	+	+	+	?	+	+	+
Friedman et al (NCT02921269)[39]	+	+	+	+	+	?	+	+
Tabernero et al (NCT02458638)[40]	+	+	+	+	+	+	+	+
Lheureux et al (NCT01693783)[41]	+	+	+	?	?	+	+	?

Figure S1. ROBINS-I tool for risk of bias.
